# A Computational Model for the AMPA Receptor Phosphorylation Master Switch Regulating Cerebellar Long-Term Depression

**DOI:** 10.1371/journal.pcbi.1004664

**Published:** 2016-01-25

**Authors:** Andrew R. Gallimore, A. Radu Aricescu, Michisuke Yuzaki, Radu Calinescu

**Affiliations:** 1 Department of Computer Science, University of York, York, United Kingdom; 2 Computational Neuroscience Unit, Okinawa Institute of Science and Technology Graduate University, Okinawa, Japan; 3 Division of Structural Biology, Wellcome Trust Centre for Human Genetics, University of Oxford, Oxford, United Kingdom; 4 Department of Physiology, Graduate School of Medicine, Keio University, Tokyo, Japan; The Krasnow Institute for Advanced Studies, UNITED STATES

## Abstract

The expression of long-term depression (LTD) in cerebellar Purkinje cells results from the internalisation of α-amino-3-hydroxy-5-methylisoxazole-4-propionic acid receptors (AMPARs) from the postsynaptic membrane. This process is regulated by a complex signalling pathway involving sustained protein kinase C (PKC) activation, inhibition of serine/threonine phosphatase, and an active protein tyrosine phosphatase, PTPMEG. In addition, two AMPAR-interacting proteins–glutamate receptor-interacting protein (GRIP) and protein interacting with C kinase 1 (PICK1)–regulate the availability of AMPARs for trafficking between the postsynaptic membrane and the endosome. Here we present a new computational model of these overlapping signalling pathways. The model reveals how PTPMEG cooperates with PKC to drive LTD expression by facilitating the effect of PKC on the dissociation of AMPARs from GRIP and thus their availability for trafficking. Model simulations show that LTD expression is increased by serine/threonine phosphatase inhibition, and negatively regulated by Src-family tyrosine kinase activity, which restricts the dissociation of AMPARs from GRIP under basal conditions. We use the model to expose the dynamic balance between AMPAR internalisation and reinsertion, and the phosphorylation switch responsible for the perturbation of this balance and for the rapid plasticity initiation and regulation. Our model advances the understanding of PF-PC LTD regulation and induction, and provides a validated extensible platform for more detailed studies of this fundamental synaptic process.

## Author Summary

Changes in synaptic strength, which can include long-term potentiation and long-term depression, are important for learning and the encoding of memories across the brain. Long-term depression (LTD), in particular, is thought to be essential for motor learning in the cerebellum, and disruption of this process, by disease or injury, can result in severe motor dysfunction. Cerebellar LTD is achieved by reducing the population of AMPA receptors at the Purkinje cell postsynaptic membrane. This population is maintained by a dynamic trafficking loop, in which AMPA receptors are continuously removed from the postsynaptic membrane by endocytosis and reinserted by exocytosis. Specific phosphorylation sites on the AMPA receptors regulate their interaction with proteins that either stabilise the receptors at the membrane or promote their trafficking. We develop a detailed bidirectional computational model of this trafficking loop and its regulation. The model shows how perturbing the trafficking balance towards AMPA receptor mobilisation and endocytosis can account for rapid induction of cerebellar LTD, and suggests mechanistic explanations for numerous features observed experimentally. This deepens our understanding of cerebellar LTD and provides a foundation for further experimental studies of this synaptic process.

## Introduction

The functional plasticity of neuronal synapses, including long-term potentiation (LTP) and long-term depression (LTD), is essential for learning and the encoding of memories [1]. The focus of this study is LTD at the parallel fibre-Purkinje cell (PF-PC) synapse in the cerebellum, which is believed to play an important role in motor learning [2–4]. This form of LTD requires [5, 6] the concurrent activation of a sufficiently large fraction of the around 175,000 excitatory *en passant* contacts made from cerebellar parallel fibres to the Purkinje cell dendritic tree [7] and of a climbing fibre comprising several thousand synaptic contacts [8]. PF-PC LTD is linked to the endocytic removal of α-amino-3-hydroxy-5-methylisoxazole-4-propionic acid receptors (AMPARs) from the Purkinje cell postsynaptic membrane [9–11]. The synaptic AMPAR population is dynamically controlled through lateral diffusion into and out of the synapse [8], and receptor endocytosis and exocytosis between the cell surface and the endosome [12]. Endosomes store the internalised AMPARs before they are directed to either reinsertion into the membrane during plasticity [2–4] or degradation [13]. The AMPAR degradation [11, 14] and *de novo* synthesis [15] provide additional regulation for the receptor population. PF-PC LTD is dependent on the increased internalisation of AMPARs relative to their reinsertion [16].

PF-PC LTD is induced by the activation of protein kinase C (PKC) [17], elevated intracellular calcium [18] and the concurrent inhibition of serine/threonine phosphatase activity [19, 20]. The mechanics of PF-PC LTD are partly controlled by two AMPAR-GluA2 subunit interacting proteins, glutamate receptor interacting protein (GRIP) and protein interacting with C kinase 1 (PICK1) [5, 6], both of which bind at the same site via their C-terminal PDZ domains [21]. The three GRIP isoforms are functionally indistinguishable [22], so we refer to them simply as GRIP. GRIP interacts with AMPARs, stabilising and clustering them both at the plasma membrane and at intracellular endosomal pools [23, 24]. This interaction prevents AMPAR trafficking [21, 23, 25], and AMPAR dissociation from GRIP is essential for the expression of PF-PC LTD [26]. AMPARs that lack the GRIP interaction are unable to stably incorporate into synapses [27].

PICK1 actively promotes AMPAR endocytosis in cerebellar Purkinje cells [6, 26, 28] and the PICK1-AMPAR interaction is indispensable for PF-PC LTD expression [6, 10, 28–30]. PICK1 also associates with the active form of PKCα [31], which phosphorylates the S880 C-terminus residue of the AMPAR-GluA2 subunit [32, 33] sustained by positive feedback mechanisms for at least 20 minutes during LTD induction [34, 35]. GluA2-S880 phosphorylation, which is elevated after the induction of LTD in hippocampal slices [36] and is required for PF-PC LTD [37], abolishes binding between GluA2 and GRIP. However, GluA2 binding to PICK1 is unaffected [38]. Dissociation of GRIP therefore allows PICK1 to bind at the same AMPAR-GluA2 site, promoting AMPAR internalisation. Disruption of the GluA2-GRIP interaction and AMPAR declustering are specifically associated with LTD induction [39]. PICK1 also interacts with GRIP and this enhances GluA2-S880 phosphorylation, possibly by directing PKCα to the GluA2 subunit [33]. The role of PICK1 in AMPAR reinsertion remains unclear, with several studies suggesting conflicting roles [21, 28, 40, 41].

Phosphorylation of the tyrosine GluA2-Y876 by Src family kinases (SFKs) negatively interferes with the GluA2-S880 phosphorylation, suggesting a regulatory role of GluA2-Y876 in LTD induction [42]. GluA2-Y876 phosphorylation levels are determined by the balance between endogenous SFK and protein tyrosine phosphatase activities. The GluA2-Y876 site is predominantly phosphorylated during basal conditions [42] and actively dephosphorylated during mGluR1-mediated LTD induction [43]. The δ2-glutamate receptor (GluD2)-associated tyrosine phosphatase, PTPMEG, actively dephosphorylates the GluA2-Y876 position *in vitro* [42], and PTPMEG-null mice display impaired motor learning and LTD [44]. By dephosphorylating the GluA2-Y876 site and hence facilitating GluA2-S880 phosphorylation, PTPMEG gates the induction of LTD in the cerebellum [42].

To gain insight into the regulation of AMPAR mobility in cerebellar LTD, we constructed a bidirectional kinetic computational model of PF-PC LTD that emphasises AMPAR trafficking as a dynamic recycling loop, and the role of GRIP, PICK1 and the relevant kinases and phosphatases in maintaining this loop. This is the first model to explicitly account for the dynamic regulation of AMPAR mobility by the interaction of the GluA2-Y876 and GluA2-S880 phosphorylation sites, now known to be a key regulatory switch for PF-PC LTD induction. Our conceptually simple model sheds light on LTD signalling beyond the well-established data showing that PF-PC LTD is dependent on PKC activation, Ca^2+^ elevation and serine/threonine phosphatase inhibition [19]. We predict that PTPMEG cooperates with PKC to drive LTD expression by gating the effect of PKC on the dissociation of AMPARs from GRIP and thus their availability for binding to PICK1 and internalisation from the postsynaptic membrane. We also show that serine/threonine phosphatase inhibition increases the degree of LTD expression, in line with experimental data [45, 46], and that SFK is not required for the induction of LTD, but negatively regulates LTD expression, as demonstrated experimentally [47]. These results advance our understanding of PF-PC LTD regulation and induction, suggest new hypotheses for experimental validation and provide a platform for further computational studies.

## Results

### Overview of the Model

**Fig 1:**
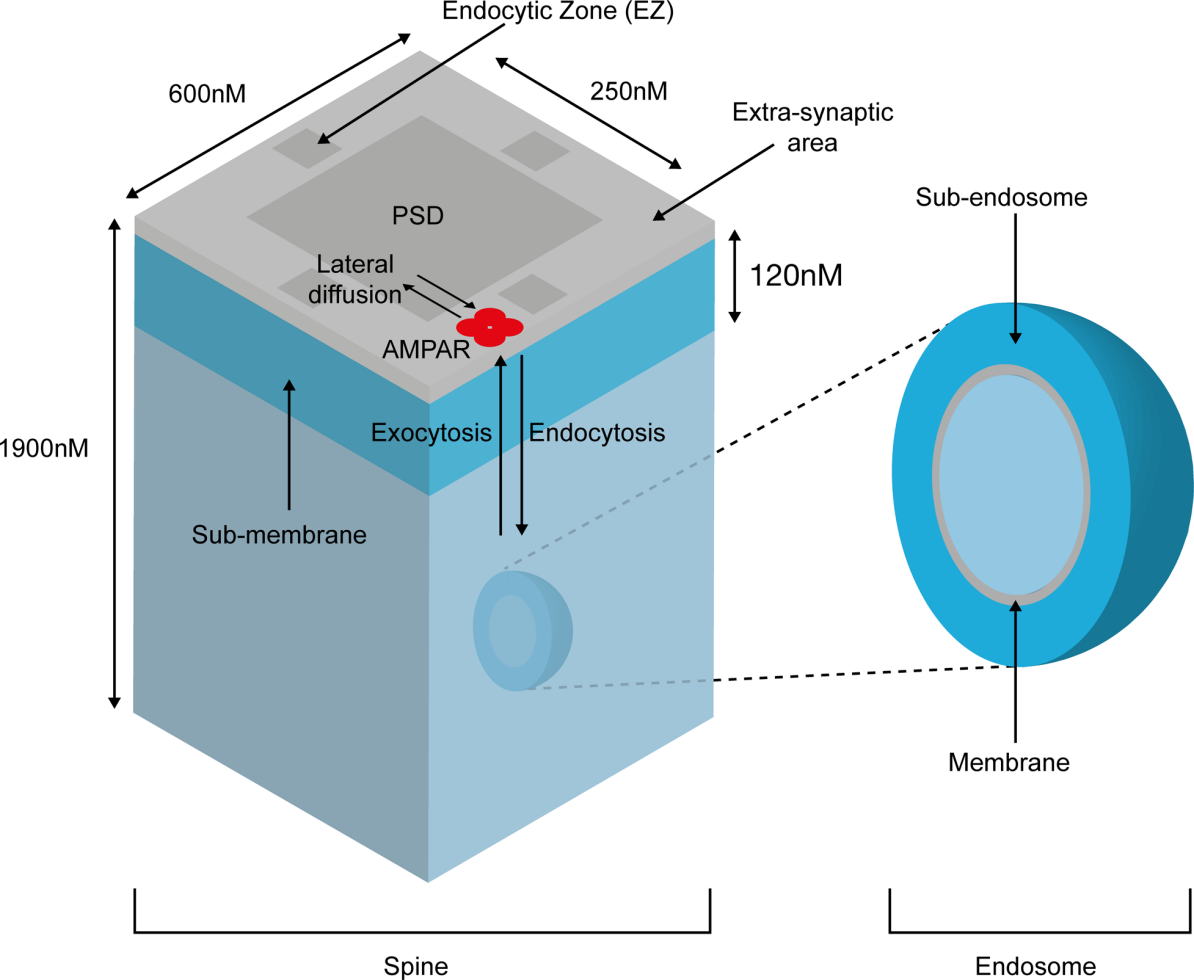
Structure of the dendritic spine model. The model contains submembrane and subendosomal compartments, with AMPAR lateral diffusion, endocytosis and exocytosis.

We model AMPARs as embedded at the cell membrane or the endosome, with all interactions with protein partners occurring in the sub-membrane and the ‘sub-endosome’ regions, respectively. These regions constitute the two main compartments of the model, and the bulk cytosol merely acts as a source/sink for smaller molecules. The sub-membrane contains three sub-compartments–the postsynaptic density (PSD), the extra-synaptic area and the endocytic zone, and AMPARs can diffuse laterally between these areas (Fig 1). The recruitment of AMPARs is a three-step process [48] comprising exocytosis at extra-synaptic areas, lateral diffusion to the PSD, and trapping by scaffold proteins (GRIP). Only AMPARs within the endocytic zone can be internalised [49, 50]. Trapping of AMPARs at the endocytic zone by dephosphorylated stargazin (TARP-γ2) is essential for LTD expression [51–53]. In line with this data, LTD is well expressed in our model only when the diffusion rate out of the endocytic zone is kept very low (<0.01s^-1^).

N-ethylmaleimide-sensitive factor (NSF) interacts with GluA2-containing AMPARs and has an essential role in the recruitment of AMPARs into the postsynaptic membrane, possibly by controlling SNARE-dependent exocytosis [54] or promoting lateral diffusion to the PSD [55, 56]. We model the potentially manifold roles of NSF by requiring that AMPARs are bound to NSF in order to undergo exocytosis [57]. As NSF disrupts the AMPAR-PICK1 interaction [58], and AMPARs bound to GRIP are not available for trafficking, only AMPARs bound to neither PICK1 nor GRIP can bind to NSF [54, 57].

We model AMPAR trafficking exclusively as a recycling loop, and LTD as a perturbation of this dynamic trafficking equilibrium. Therefore, we do not consider *de novo* synthesis and degradation of AMPARs, whose inclusion is likely to occlude the effect of the phosphorylation switch on AMPAR mobility and LTD expression. Furthermore, degradation of internalised AMPARs does not have functional consequences for the regulation of LTD [59], although the regulation of AMPAR recycling is essential for determining the degree of LTD expression [60].

**Fig 2:**
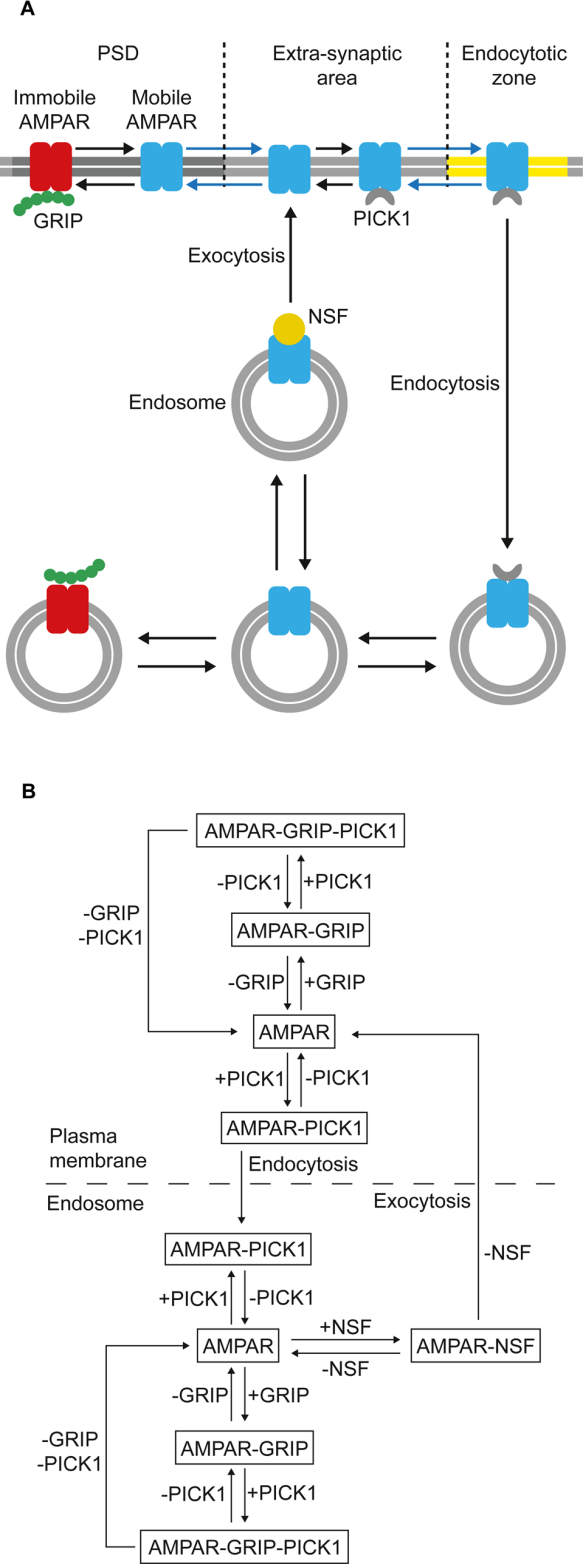
AMPAR trafficking in the model. (A) Schematic representation of AMPAR trafficking between the postsynaptic plasma membrane and endosome used in the model. (B) Detailed bidirectional trafficking pathway showing the interactions between AMPARs, GRIP, PICK1 and NSF.

Many published LTD models are unidirectional and measure LTD expression in terms of AMPAR internalization only, or even simply by the level of AMPAR phosphorylation [61]. This simplifies the modeling strategy but neglects the importance of the endocytosis-exocytosis balance in regulating the cell surface AMPAR population and the dynamic nature of AMPAR recycling. A sophisticated recent stochastic model of cerebellar LTD [35] does account for exocytosis of AMPARs, but disregards all other interactions within the intracellular compartment that are important in regulating AMPAR mobility and reinsertion. Our trafficking pathway is a bidirectional kinetic model (Fig 2) that emphasises AMPAR trafficking as a dynamic recycling loop. As with other models of LTD, we measure LTD expression purely in terms of the reduction of the postsynaptic membrane AMPAR population [35, 61], although additional mechanisms, such as AMPAR desensitisation, may also play a minor role in the biological system [62].

**Fig 3:**
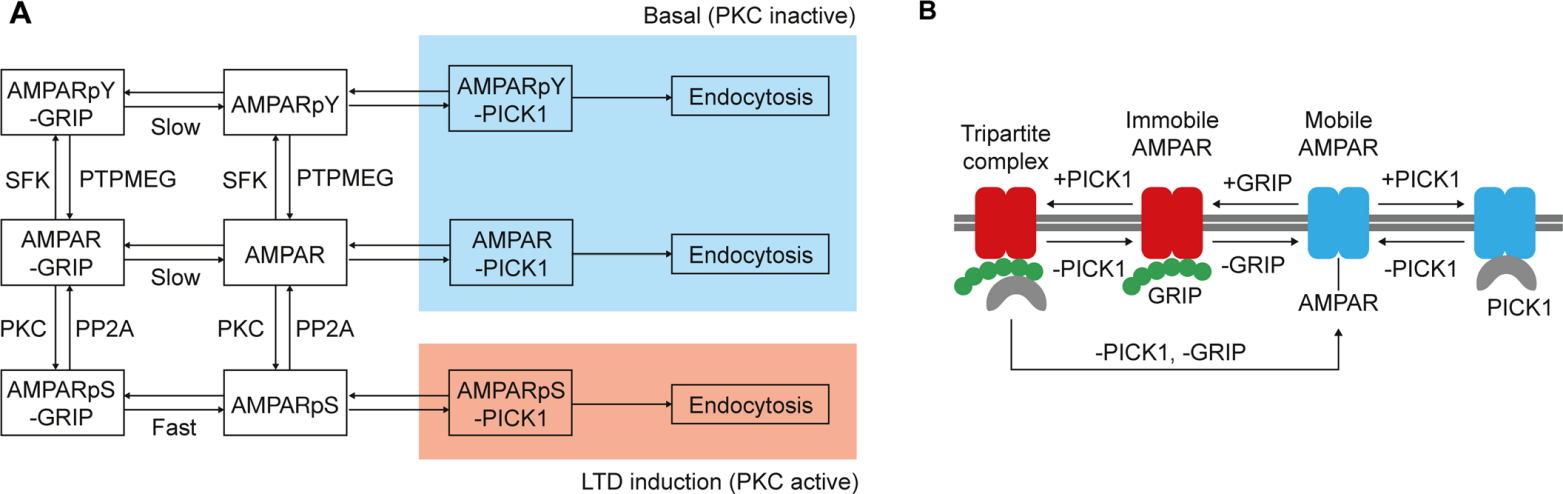
Interconversion of the AMPAR phosphorylation states in the model. (AMPApY = GluA2-Y876-phosphorylated AMPA receptor; AMPApS = GluA2-S880-phosphorylated AMPAR). (A) Influence of AMPAR phosphorylation state on GRIP and PICK1 interactions and trafficking during basal and LTD induction conditions. (B) Interactions between AMPAR and GRIP/PICK1, showing the formation of a tripartite complex.

Unique to our model, the dissociation from GRIP, and the mobilisation and availability of AMPARs for trafficking between compartments are regulated by the mutually exclusive phosphorylation of the GluA2-S880 and GluA2-Y876 sites (Fig 3A) [42]. GluA2-S880 is phosphorylated by PKC and dephosphorylated by PP2A, while GluA2-Y876 is phosphorylated by SFKs [42] and dephosphorylated by PTPMEG [42]. Phosphorylation of the GluA2-S880 site abolishes the interaction between the AMPAR and GRIP, allowing PICK1 to bind. PICK1 can also associate with GRIP directly to form a tripartite complex (Fig 3B). The other interactions within the model are detailed in the Methods section.

### PTPMEG gates PKC-driven AMPAR mobilisation and internalisation

**Fig 4:**
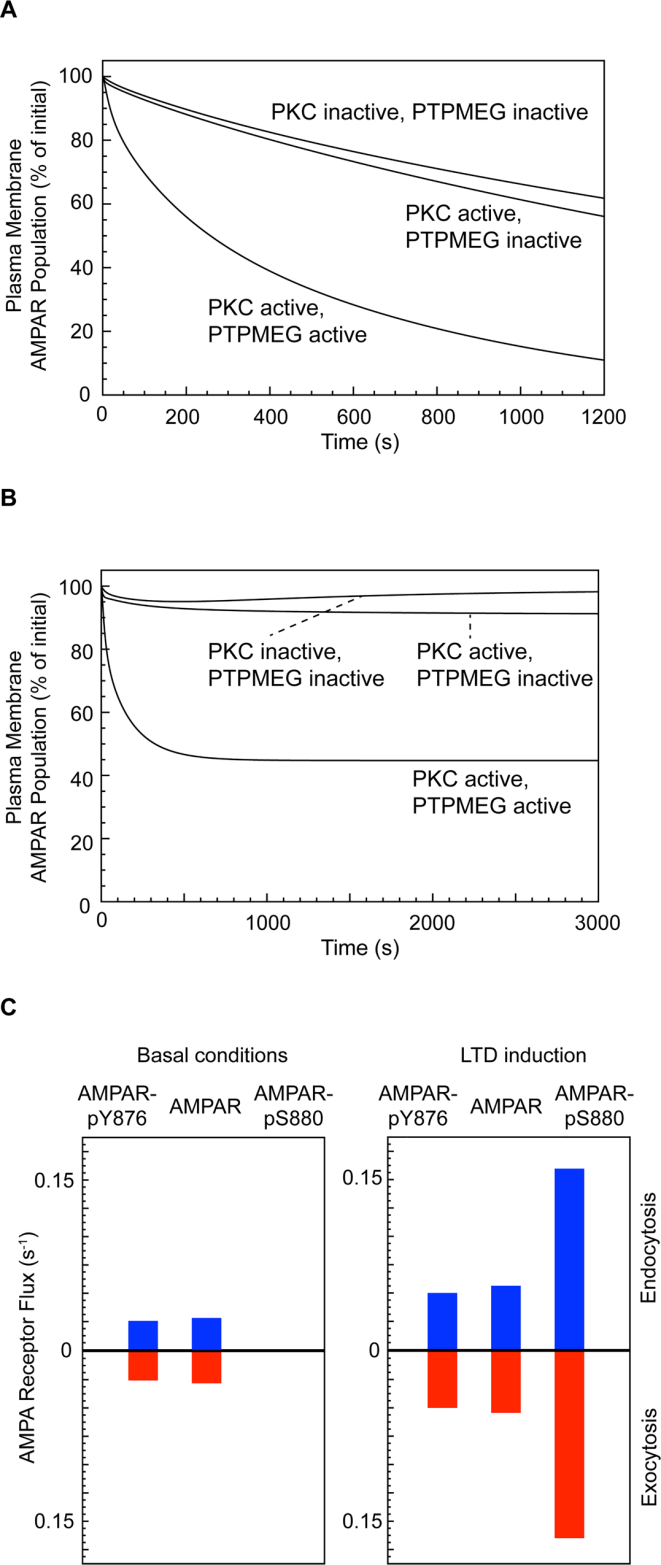
AMPAR trafficking under basal conditions and during LTD induction. (Total AMPAR plasma membrane population includes both phosphorylated, at either GluA2-Y876 or GluA2-S880, and unphosphorylated AMPARs.) (A) Effect of PKC and PTPMEG on AMPAR plasma membrane population when exocytosis is blocked. (B) Effect of PTPMEG on LTD induction in the complete model. (C) AMPAR flux across the plasma membrane under basal and LTD induction conditions.

To observe the effect of PKC and PTPMEG on the endocytic rate alone, we initially selectively blocked exocytosis. Under basal conditions, when PKC is inactive, approximately 125 AMPARs populate the PSD [63] and around 40% of these are estimated to be internalised within 20 minutes [57]. When PKC is activated in the absence of active PTPMEG, the average rate of endocytosis is only slightly elevated relative to basal conditions (44% of AMPARs internalised over 20 minutes with activated PKC, versus 38% when PKC is inactive) (Fig 4A). However, activation of PKC in the presence of active PTPMEG increases the internalisation rate 2-fold above that generated by activated PKC alone, with 89% of AMPARs being internalised over 20 minutes. This suggests that the role of PTPMEG is to gate the effect of active PKC in promoting AMPAR dissociation from GRIP and subsequent internalisation. The result is in agreement with experimental data, which shows that elevated PKC alone does not increase the AMPAR internalisation rate in cerebellar Purkinje cells [64].

### During LTD induction, the S880-phosphorylated form of AMPAR is internalised

According to our LTD model, under basal conditions, the AMPARs trafficked between the cell surface and endosome are predominantly the unphosphorylated and GluA2-Y876-phosphorylated forms. During LTD induction, we expected a shift towards internalisation of the GluA2-S880-phosphorylated form of the receptor as PKC is activated. We reinstated exocytosis and measured the flow of the three different forms of AMPAR (unphosphorylated, GluA2-Y876-phosphorylated and GluA2-S880-phosphorylated) between the plasma membrane and endosomal compartments and vice versa in 3000-second simulations of the system under basal conditions, and during PKC-induced LTD (Fig 4B). Under basal conditions, the cell surface AMPAR population remained stable and only the unphosphorylated form of AMPAR and the GluA2-Y876 phosphorylated form were internalised, each being trafficked at a rate of 0.03–0.04 receptors per second, equally in both directions. When PKC was activated in the presence of PTPMEG, the cell surface AMPAR population declined to 44% of its initial number over around 1000 seconds. This was followed by a steady state during which mainly the GluA2-S880-phosphorylated form of AMPAR was internalised, with 0.16 of these receptors being trafficked per second in both directions, in addition to a small number (0.04–0.06 per second for each) of the unphosphorylated and GluA2-Y876 phosphorylated forms of the receptor (Fig 4C).

### LTD is not induced in a PTPMEG-null system

When PKC is inactive, the cell surface AMPAR population remains stable, both in the presence and absence of PTPMEG. To analyse the effect of PKC activation, we ran 3000-second model simulations comprising 1000 seconds under basal conditions, followed by a step function activation of PKC that was maintained for the remaining 2000 seconds. This represents the approximate period for which PKC activation is maintained by a positive feedback mechanism during LTD induction, in line with experimental data [35]. As late phase effects maintain LTD after the PKC activation window, we do not consider deactivation of PKC or the maintenance of LTD after this time.

**Fig 5:**
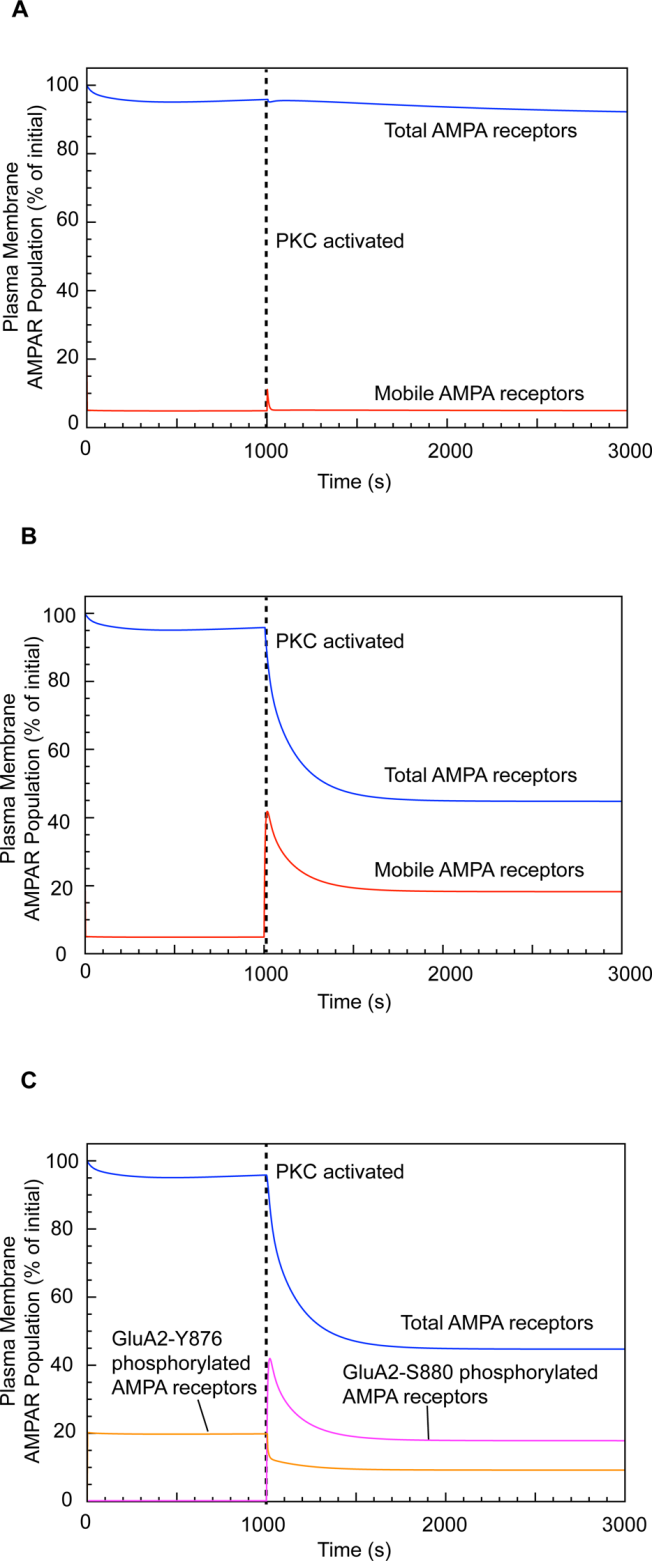
Effect of PTPMEG on LTD expression. (Total AMPAR plasma membrane population includes both phosphorylated, at either GluA2-Y876 or GluA2-S880, and unphosphorylated AMPARs.) (A) LTD expression in a PTPMEG-null system. (B) LTD expression in a PTPMEG-active system. (C) Changes in AMPAR phosphorylation state during LTD induction.

In the absence of PTPMEG, and in agreement with experimental results [42, 44], the activation of PKC does not result in a marked inward trafficking of plasma membrane AMPARs, with the cell surface population of AMPARs only falling to 92% of baseline when PKC is activated. Furthermore, there is no increase in the number of mobile AMPARs (i.e. not bound to GRIP), with fewer than 6% of the AMPARs being mobile during the PKC activation period, as during basal conditions (Fig 5A).

### PTPMEG gates LTD in the presence of active PKC

When PTPMEG is present, the activation of PKC leads to an immediate increase in the average percentage of cell surface AMPARs that are mobile from ~6% to ~18% (Fig 5B). This demonstrates cooperation between PKC and PTPMEG to mobilise the cell surface AMPARs for trafficking. Neither PKC activation nor PTPMEG alone is capable of eliciting LTD. Both enzymes are required concurrently, as suggested by experimental data demonstrating that LTD expression in cerebellar Purkinje cells requires PTPMEG activity [42]. The increase in mobile AMPARs during the PKC activation window triggers a decline in the cell surface AMPAR population towards a steady state as endocytosis dominates the trafficking dynamics (Fig 5B).

Experiments have shown that the population of GluA2-Y876-phosphorylated AMPARs declines during LTD induction [42], with the GluA2-S880-phosphorylated form increasing concurrently [36], as plasma membrane AMPARs are mobilised and internalised. Our simulations replicate and quantify this effect (Fig 5C). Under basal conditions in our model, approximately 20% of membrane AMPARs are GluA2-Y876 phosphorylated, with none of the receptors phosphorylated at the GluA2-S880 site. However, immediately upon PKC activation, the population of GluA2-S880-phosphorylated AMPARs increased to 18% of the total PSD AMPAR population, and this was maintained throughout the PKC activation window. Comparable with experimental observations [42], the population of GluA2-Y876 phosphorylated receptors declined from 20% to 9% upon PKC activation (Fig 5C).

### Phosphatase inhibition enhances AMPAR mobilisation and LTD expression

**Fig 6:**
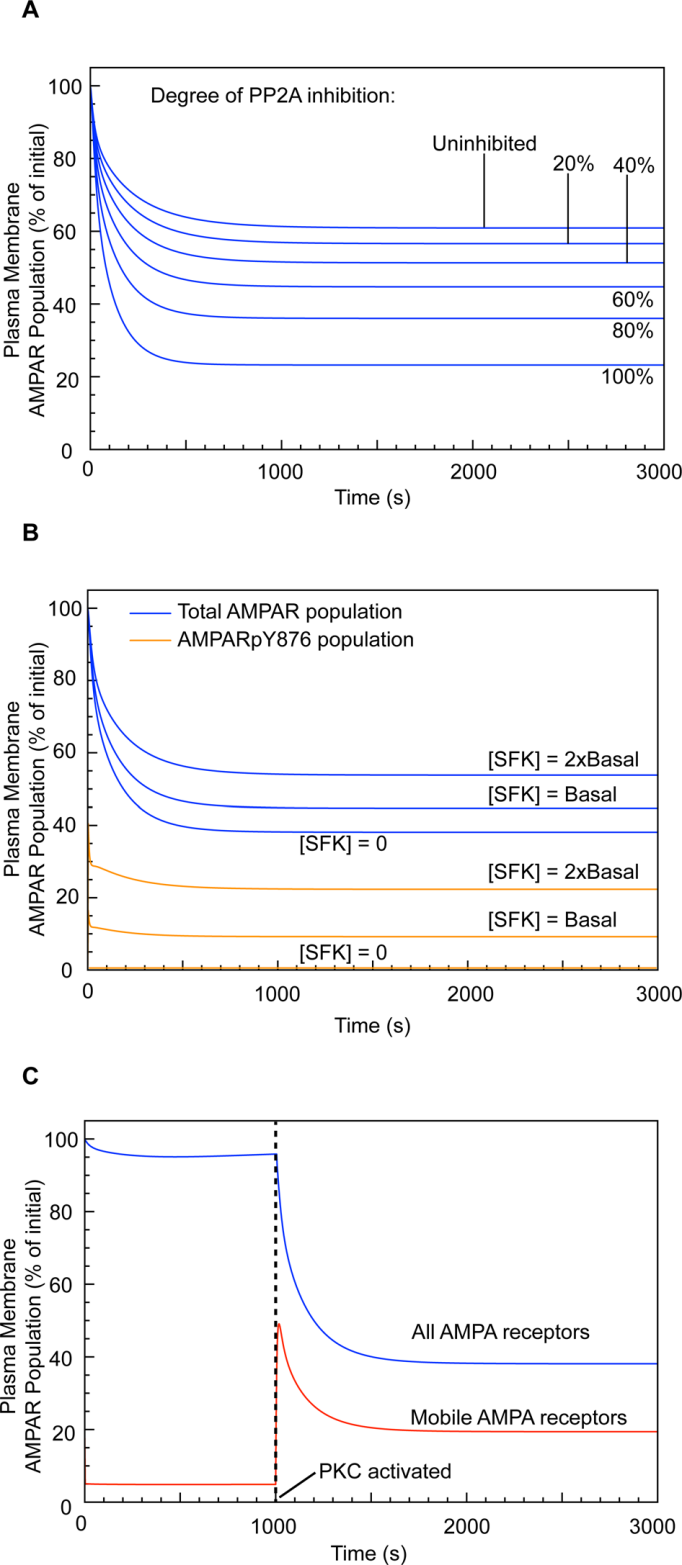
Effect of PP2A inhibition and SFK on LTD expression. (A) Effect of varying PP2A inhibition on LTD expression. (B) Effect of increased SFK concentration on LTD expression (only 2 concentrations and SFK-null shown–see Table 2 for the complete set of results). (C) LTD expression in a PTPMEG-null, SFK-null system (equivalent to GluA2-Y876F expression in the in vitro system).

It is well established that the inhibition of serine/threonine phosphatase activity accompanies LTD induction [19, 45, 65]. However, whether such inhibition is essential for LTD induction or merely augments is not understood. To study the effects of phosphatase inhibition on LTD induction, we performed simulations for PP2A concentrations ranging between 0–100% (Fig 6 and Table 1). Increasing phosphatase inhibition results in a corresponding increase in the degree of LTD achieved. Without PP2A inhibition, only a 39% reduction in cell surface AMPAR population is achieved after 20 minutes, rising to 77% reduction with 100% PP2A inhibition. This result is comparable to experimental results showing up to a 65% reduction in excitatory postsynaptic current amplitude in cerebellar Purkinje cells using PP2A inhibitors [45], and suggests that tuning of phosphatase inhibition could regulate the degree of depression achieved during LTD.

**Table 1:** Effect of PP2A inhibition on LTD expression.

PP2A Inhibition (%)	Plasma membrane (PSD) AMPAR population at 20 min relative to baseline (%)
0	61
20	57
40	51
60	44
80	36
100	23

### SFK activity negatively regulates the degree of LTD expression, but is not required for LTD induction

SFKs selectively phosphorylate the Y876 site of the AMPAR GluA2 subunit [42]. Under basal conditions, phosphorylation at this position limits GluA2-S880 phosphorylation. By allowing GRIP to bind, this stabilises the AMPARs at the cell surface or endosomal membrane. Active PTPMEG dephosphorylates GluA2-Y876, enabling GluA2-S880 phosphorylation and hence the dissociation of the AMPAR from GRIP and its mobilisation for trafficking. We performed simulations under standard LTD induction conditions, in the absence of SFKs, and with increasing SFK concentrations up to 5-fold greater than the basal concentration. Removing SFKs from the system slightly enhanced LTD expression, with 38% of cell surface (PSD) AMPARs remaining after 20 minutes, compared to 44% for the wild-type conditions. Increasing concentrations of SFK caused a proportional decrease in the magnitude of the LTD response, which was directly related to the degree of GluA2-Y876 phosphorylation (Fig 6B and Table 2).

**Table 2:** Effect of SFK concentration on LTD expression.

SFK concentration relative to wild-type	Plasma membrane (PSD) AMPAR population at 20 min relative to baseline (%)	Remaining plasma membrane (PSD) GluA2-Y876 phosphorylated AMPAR population at 20 min (%)
SFK null	38	0
SFK wild-type	44	9
SFK 2x	54	22
SFK 5x	84	66

This result is in agreement with experimental studies showing that SFK negatively regulates cerebellar LTD expression [47], although it appears to contradict earlier studies showing that SFKs are essential for LTD expression [66, 67], with SFK inhibitors abolishing LTD. However, the broad-spectrum tyrosine kinase inhibitors used in these studies (i.e. genistein and lavendustin A) are likely to affect kinases other than SFKs [42]. If a more specific SFK inhibitor is used to reduce tyrosine (GluA2-Y876) phosphorylation, LTD induction in cerebellar Purkinje cells is unaffected [42, 47], in agreement with our results. It should be noted that, *in vivo*, SFKs act on a broad range of substrates and, as such, their effect on AMPAR trafficking, both directly and indirectly, could be more complex than indicated by our model. However, the effect of SFK at the GluA2-Y876 phosphorylation site is sufficient to explain current experimental data.

### LTD is rescued by GluA2-Y876F expression in a PTPMEG-null system

Knockout of PTPMEG or the PTPMEG-interacting GluD2 abrogates LTD [42] by preventing AMPAR mobilisation. Expression of the mutant subunit, GluA2-Y876F, which cannot be tyrosine phosphorylated, rescues LTD in GluD2-null Purkinje cells [42]. We replicated this result by blocking GluA2-Y876 phosphorylation. Under these conditions, even when PTPMEG was knocked out, LTD was fully expressed (Fig 6C). This demonstrates the central role of GluA2-Y876 phosphorylation in the regulation of AMPAR mobility. The role of SFK activity thus appears to be in limiting AMPAR mobilisation under basal conditions, as well as being an active regulator of PF-PC LTD.

## Discussion

The AMPAR population at the Purkinje cell postsynaptic membrane is part of a continuous dynamic recycling loop. Even when the population is stable, under basal conditions, 90% of the internalised AMPARs are returned to the cell surface within 60 minutes [14]. It is this dynamism that ensures a rapid response to perturbation. Modelling both directions of AMPAR trafficking simultaneously is therefore essential for the accurate study of plasticity. Furthermore, a number of proteins and signalling pathways that regulate receptor internalisation may also affect reinsertion. Consequently, any LTD model that considers only the regulation of internalisation will necessarily be incomplete and may even produce misleading data. In a study of the effects of synaptic activity on AMPAR trafficking in cultured cortical neurons [14], manipulating the rate of AMPAR internalisation–using tetrodotoxin and picrotoxin–had no effect on the size of the cell surface AMPAR population, as the reinsertion rate was similarly affected. It is thus clear that the regulatory mechanisms controlling AMPAR internalisation overlap with those controlling reinsertion. As such, AMPAR trafficking is best described as a unified recycling loop rather than two separate processes.

The balance of kinase and phosphatase activity within cerebellar Purkinje cells is exquisitely poised to allow the AMPAR population to be stabilised at the cell surface and endosome, and yet rapidly mobilised for trafficking during LTD induction. Our simulations show that the GluA2-Y876 and GluA2-S880 phosphorylation sites together act as a ‘master switch’ both for the induction of PF-PC LTD and the regulation of its magnitude. Whilst PTPMEG acts as an overall facilitator of LTD induction, by gating the dissociation of AMPARs from their GRIP anchors, PP2A and SFK activity can tune the degree of depression achieved. This is an important insight that clarifies, and provides a straightforward molecular mechanism for, the role of kinase and phosphatase activity in LTD regulation. Experimental studies have established that PP2A inhibition enhances LTD expression [45], and that SFK activity negatively regulates it [47], in agreement with our simulations. Furthermore, Endo *et al* [68] produced mutant mice lacking the gene coding for G-substrate, a potent inhibitor of PP2A [69]. Surprisingly, the consequent elevated PP2A levels did not abolish LTD in cerebellar Purkinje cells. Our model explains this result, and demonstrates that PP2A inhibition regulates the magnitude of LTD achieved, but is not required for LTD induction (Table 3).

**Table 3:** Summary of simulation results compared to corresponding experimental results.

	Degree of LTD Achieved (% depression from baseline)		
Experiment	Experimental data	Simulation data	Comments
LTD induction (wild-type)	45% [70]	39–77% (dependent on degree of PP2A inhibition)	The degree of LTD achieved in experiments is dependent on the induction protocol and varies between labs.
	38% [42]		
PTPMEG null	5% (vs 32% wild-type) [71]	8% (vs 56% wild-type)	[71] deleted GluRδ2 rather than PTPMEG. The profound effect on LTD expression may result only partly from disruption of the GluRδ2-PTPMEG interaction.
	23% (vs 38% wild-type) [44]		
PP2A inhibition	65% [45]	56–77% (60–100% PP2A inhibition)	The degree of PP2A inhibition achieved is not reported in [45].
G-substrate null (PP2A inhibition pathway blocked)	17% (vs 22% wild-type) [68]	39% (vs 49% with 40% PP2A inhibition)	A modest reduction in LTD magnitude is achieved by blocking PP2A inhibition.
SFK null	No effect compared to wild-type [42]	62% (vs 56% wild-type)	Only a modest increase in LTD magnitude is obtained by removing SFK from the model.
SFK elevated	9% (vs 33% control) [47]	16% with [SFK] increased 5x relative to [basal] (vs 56% control)	The Purkinje cell concentration of SFK achieved is not reported by [47].

Although the orphan glutamate receptor δ2 (GluD2) is indispensable for PF-PC LTD expression [72], its specific role remains unclear. However, by binding to and potentially activating PTPMEG, it may concentrate this phosphatase at the plasma membrane and thus facilitate the selective mobilisation of cell surface AMPARs. Whilst GluD2 is only expressed in cerebellar Purkinje cells, several brain regions express GluD1 [73], which may function in a similar manner by binding and/or leading to PTPMEG activation, making this phosphatase a more global regulator of plasticity than currently known. Furthermore, PTPMEG has been shown to bind the NR2A subunit of NMDA receptors [74], which could also support this function.

The signalling pathways regulating synaptic plasticity are complex, both in terms of the number of signalling species involved and their spatiotemporal dynamics. This makes any bidirectional model of trafficking challenging to construct and implement, but essential for generating realistic data. Our model achieves this, is able to replicate a wide range of experimental observations of cerebellar parallel fibre-Purkinje cell LTD, sheds light on their underpinning mechanisms and provides a sound foundation for additional simulation experiments and for more detailed models of synaptic plasticity processes. Furthermore, our model is the first to explore the role of this type of mutually-exclusive phosphorylation switch, which is similar to switches found in other important systems, including receptors controlling insulin response [75], and NMDA receptor function [76].

## Methods

### Model implementation

The model was implemented in the well-established and validated open-source biochemical network simulator COPASI [77, 78], using kinetic parameters obtained from the literature (see supplementary information S1 Table for details). We used deterministic simulation to efficiently and accurately establish the average system behaviour for a wide range of scenarios and parameter ranges [79]. These simulations were performed using the COPASI built-in LSODA (Livermore Solver for Ordinary Differential Equations) solver, with particle number to concentration conversions performed by COPASI. Model can be found in S1 Model.

### Model compartments

The model contains two compartments (Fig 1). The sub-membrane compartment comprises the volume of cytosol directly below the plasma membrane to a distance of 120nm [80], and consists of three sub-compartments: postsynaptic density (PSD), endocytic zone (EZ) and extra-synaptic area. The sub-endosome compartment is assumed to occupy the same volume as the sub-membrane. As AMPARs are entirely membrane-bound, they are concentrated in these regions and hence all of the key reactions occur here. The bulk cytosol, which is not explicitly modelled, merely acts as a source/sink for species that are distributed throughout the dendritic spine. Thus, when a species, such as GRIP or PICK1, binds to an AMPAR, it is immediately replaced, by diffusion, by a spare from the bulk cytosol. This approach is supported by experimental and modelling data suggesting that AMPAR scaffolds are never saturated [12]. However, we also produced an alternative model in which GRIP and PICK1 numbers were finite. This model produced results qualitatively the same as those produced with the model used in our paper. The alternative model, together with representative results, is included in the supplementary information S2 Model and S1 Fig.

The complete set of model reactions is summarised in Table 4 and is described below. Except where explicitly stated, these reactions occur in each compartment of the model, between species from the populations in that compartment.

**Table 4:** Model reactions (rate parameters provided in S1 Table).

Reaction number	Reaction	Rate
**AMPAR Binding Interactions at PSD and the Endosome** ^†^		
1	AMPAR + GRIP → AMPAR-GRIP	k.ampar-grip.on
2	AMPAR-GRIP → AMPAR + GRIP^1^	k.ampar-grip.off^1^
	AMPARpS-GRIP → AMPARpS + GRIP^2^	k.ampar-grip.offpS^2^
3	AMPAR + PICK1 → AMPAR-PICK1	k.ampar-pick.on
4	AMPAR-PICK1 → AMPAR + PICK1	k.ampar-pick.off
5	AMPAR + PICK1-PKC* → AMPAR-PICK1-PKC*	k.ampar-pick.on*
6	AMPAR-PICK1-PKC* → AMPAR + PICK1-PKC*	k.ampar-pick.off
7	AMPAR-GRIP + PICK1 → AMPAR-GRIP-PICK1	k.grip-pick.on
8	AMPAR-GRIP-PICK1 → AMPAR-GRIP + PICK1	k.grip-pick.off
9	AMPAR-GRIP + PICK1-PKC* → AMPAR-GRIP-PICK1-PKC*	k.grip-pick.on
10	AMPAR-GRIP-PICK1-PKC* → AMPAR-GRIP + PICK1-PKC*	k.grip-pick.off
11	AMPAR-GRIP-PICK1 → AMPAR + GRIP + PICK1	k.ampar-grip.off
12	AMPAR-GRIP-PICK1-PKC* → AMPAR + GRIP + PICK1-PKC*	k.ampar-grip.off
13	AMPAR + NSF → AMPAR-NSF (endosome only)	k.ansf.on
14	AMPAR-NSF → AMPAR + NSF (endosome only)	k.ansf.off
**AMPAR Lateral Diffusion (between PSD and extra-synaptic area, X)**		
15	AMPAR → AMPAR(X)	k.diff.psd-x
16	AMPAR-PICK1 → AMPAR-PICK1(X)	k.diff.psd-x
17	AMPAR-PICK1-PKC* → AMPAR-PICK1-PKC*(X)	k.diff.psd-x
18	AMPAR(X) → AMPAR	k.diff.x-psd
19	AMPAR-PICK1(X) → AMPAR-PICK1	k.diff.x-psd
20	AMPAR-PICK1-PKC*(X) → AMPAR-PICK1-PKC*	k.diff.x-psd
**AMPAR Lateral Diffusion (between extra-synaptic area, X, and endocytic zone, EZ)**		
21	AMPAR(X) → AMPAR(EZ)	k.diff.x-ez
22	AMPAR-PICK1(X) → AMPAR-PICK1(EZ)	k.diff.x-ez
23	AMPAR-PICK1-PKC*(X) → AMPAR-PICK1-PKC*(EZ)	k.diff.x-ez
24	AMPAR(EZ) → AMPAR(X)	k.diff.ez-x
25	AMPAR-PICK1(EZ) → AMPAR-PICK1(X)	k.diff.ez-x
26	AMPAR-PICK1-PKC*(EZ) → AMPAR-PICK1-PKC*(X)	k.diff.ez-x
**AMPAR Endocytosis and Exocytosis**		
27	AMPAR-PICK1(EZ) → AMPAR-PICK1(endosome)	k.endo
28	AMPAR-PICK1-PKC*(EZ) → AMPAR-PICK1-PKC*(endosome)	k.endo
29	AMPAR-NSF → AMPAR(X)	k.exo
**Activation/inactivation of PKC**		
30	PKC → PKC*	pkc.act
31	PKC* → PKC	pkc.deact
**PICK-PKC Interactions**		
32	PICK1 + PKC* → PICK1-PKC*	k.pick-pkc.on
33	PICK1-PKC* → PICK1 + PKC*	k.pick-pkc.off
**PKC Phosphorylation at GluA2-S880**		
34	AMPAR + PKC* → AMPARpS + PKC*	kcat.pkc, km.pkc
35	AMPAR-GRIP + PKC* → AMPARpS-GRIP + PKC*	kcat.pkc, km.pkc
36	AMPAR-PICK1 + PKC* → AMPApS-PICK1 + PKC*	kcat.pkc, km.pkc
37	AMPAR-GRIP-PICK1 + PKC* → AMPARpS-GRIP-PICK1 + PKC*	kcat.pkc, km.pkc
38	AMPAR-PICK1-PKC* → AMPARpS-PICK1-PKC*	kcat.pkc
39	AMPAR-GRIP-PICK1-PKC* → AMPARpS-GRIP-PICK1-PKC*	kcat.pkc
**AMPARpS(880) Dephosphorylation**		
40	AMPARpS + PP2A → AMPAR + PP2A	kcat.pp2a, km.pp2a
41	AMPARpS-GRIP + PP2A → AMPAR-GRIP + PP2A	kcat.pp2a, km.pp2a
42	AMPARpS-PICK + PP2A → AMPAR-PICK + PP2A	kcat.pp2a, km.pp2a
43	AMPARpS-PICK-PKC* + PP2A → AMPAR-PICK-PKC* + PP2A	kcat.pp2a, km.pp2a
44	AMPARpS-GRIP-PICK + PP2A → AMPAR-GRIP-PICK + PP2A	kcat.pp2a, km.pp2a
45	AMPARpS-GRIP-PICK-PKC* + PP2A → AMPAR-GRIP-PICK-PKC* + PP2A	kcat.pp2a, km.pp2a
**SFK Phosphorylation at GluA2-Y876**		
46	AMPAR + SFK → AMPARpY + SFK	kcat.sfk, km.sfk
47	AMPAR-GRIP + SFK → AMPARpY-GRIP + SFK	kcat.sfk, km.sfk
48	AMPAR-PICK + SFK → AMPARpY-PICK + SFK	kcat.sfk, km.sfk
49	AMPAR-PICK-PKC* + SFK → AMPARpY-PICK-PKC* + SFK	kcat.sfk, km.sfk
50	AMPAR-GRIP-PICK1 + SFK → AMPARpY-GRIP-PICK1 + SFK	kcat.sfk, km.sfk
51	AMPAR-GRIP-PICK1-PKC* + SFK → AMPARpY-GRIP-PICK1-PCK* + SFK	kcat.sfk, km.sfk
**AMPARpY(876) Dephosphorylation**		
52	AMPARpY + PTPMEG → AMPAR + PTPMEG	kcat.ptpmeg, km.ptpmeg
53	AMPARpY-GRIP + PTPMEG → AMPAR-GRIP + PTPMEG	kcat.ptpmeg, km.ptpmeg
54	AMPARpY-PICK1 + PTPMEG → AMPAR-PICK1 + PTPMEG	kcat.ptpmeg, km.ptpmeg
55	AMPARpY-PICK1-PKC* + PTPMEG → AMPAR-PICK1-PKC* + PTPMEG	kcat.ptpmeg, km.ptpmeg
56	AMPARpY-GRIP-PICK1 + PTPMEG → AMPAR-GRIP-PICK1 + PTPMEG	kcat.ptpmeg, km.ptpmeg
57	AMPARpY-GRIP-PICK1-PKC* + PTPMEG → AMPAR-GRIP-PICK1-PKC* + PTPMEG	kcat.ptpmeg, km.ptpmeg

† Barring reaction 3 (dissociation of AMPARpS880 from GRIP), ‘AMPAR’ denotes *any* of AMPAR, AMPARpS880 or AMPARpY876 that can participate in each reaction.

^1^rate for AMPAR species other than AMPARpS880

^2^rate for AMPARpS880

### AMPAR binding interactions (Table 4, Reactions 1–14)

AMPARs exist freely or associated with GRIP or with PICK1, forming an AMPAR-GRIP or AMPAR-PICK1 complex, respectively (Table 4, Reactions 1–6). PICK1 may associate with the GRIP of an AMPAR-GRIP complex and thus a tripartite complex, AMPAR-GRIP-PICK1, can form (Reactions 7–12). A dimeric GRIP-PICK1 complex is not considered, as preliminary experiments showed that it had no effect on the outcome of the simulations. The GRIP populations at the PSD and the endosome interact with AMPARs identically, anchoring the AMPAR to the PSD and the endosomal compartment, respectively [81]. AMPAR-GRIP interactions are not considered in the extra-synaptic area or the endocytic zone. PICK1 is a calcium sensor and the AMPAR-PICK1 binding rate increases 4-fold in the presence of a high calcium concentration [26], as during PF-PC LTD induction. Endosomal AMPARs can also associate with NSF, but only when not associated with either GRIP or PICK1 (Reactions 13 and 14). All binding interactions are assumed to occur with mass action kinetics.

### AMPAR lateral diffusion (Table 4, Reactions 15–26)

AMPARs not bound to GRIP can diffuse laterally, in both directions, between the PSD and the extra-synaptic area (Reactions 15–20), and between the extra-synaptic area and the endocytic zone (Reactions 21–26). The rate constant for diffusion from one area to another is calculated as the ratio between the diffusion coefficient [12, 48] and the area of the sub-compartment [35].

### AMPAR endocytosis and exocytosis (Table 4, Reactions 27–29)

To undergo endocytosis (Reactions 27 and 28), a GRIP-bound plasma membrane AMPAR must detach from GRIP and bind to PICK1. Furthermore, only AMPARs at the EZ can be internalised. AMPARs can only undergo exocytosis (Reaction 29) when NSF is bound to the receptor, with AMPARs being reinserted into the extra-synaptic area. As we do not consider AMPAR-NSF interactions within the plasma membrane, AMPARs are assumed to detach from NSF when exocytosis occurs.

### AMPAR phosphorylation (Table 4, Reactions 30–55)

We adopt a simple switch for activating and deactivating PKC (Reactions 30 and 31), in line with both experimental data [34] and computational simulations [35], which show that positive feedback mechanisms maintain PKC activity for the duration of early LTD induction (at least 20 minutes). PKC can exist freely in the cytoplasm or, when in its active form (PKC*), combined in a reversible complex with PICK1 (Reactions 32 and 33).

PKC* phosphorylates AMPAR at the GluA2-S880 site to generate AMPApS(880) (Reactions 34–37). The PICK1-PKC* complex can also phosphorylate the GluA2-S880 site. Once PICK-PKC* is bound to AMPAR, phosphorylation is assumed to occur at the turnover rate for PKC* (Reactions 38 and 39). The phosphorylation of GluA2-S880 reduces the affinity of the AMPAR for GRIP, as reflected by an increase in the AMPApS-GRIP unbinding rate (Reaction 2) [35]. The AMPAR GluA2-S880 site is dephosphorylated by PP2A, which we assume constitutively active and inhibited (60%) during LTD induction (Reactions 40–45). AMPAR is phosphorylated by SFKs at the GluA2-Y876 site to generate AMPApY(876) (Reactions 46–51). Dephosphorylation of GluA2-Y876 is performed by PTPMEG (Reactions 52–57).

All phosphorylation and dephosphorylation reactions are assumed to occur with Michaelis-Menten kinetics.

### Simulation of basal conditions and LTD induction

Experimentally, under basal conditions, the majority of AMPARs are unphosphorylated [42]. In line with experimental data [63], the system was initially populated with 125 submembrane AMPARs and 125 sub-endosome AMPARs, all unphosphorylated. The kinetics of PTPMEG were calibrated such that the proportion of AMPARs phosphorylated at GluA2-Y876 was consistently approximately 25%, in line with experimental data [42]. However, simulations using alternative initial AMPAR populations–increasing the proportion of GluA2-Y876-phosphorylated AMPARs, for example–did not affect the results obtained, either qualitatively or quantitatively.

Basal conditions were defined as corresponding to PKC inactive, PP2A uninhibited and AMPAR trafficking calibrated by setting the endocytosis rate such that approximately 40% of receptors were internalised over a 20-minute period when exocytosis was selectively blocked [57]. The exocytosis rate was set such that it balanced endocytosis under basal conditions.

When simulating LTD induction, PKC was activated and PP2A was inhibited by 60% throughout the simulation. This inhibition was modelled by removing 60% of the PP2A from the model. For time course simulations, a step function was used to activate PKC (Table 4, Reactions 31 and 32) after allowing the simulation to run for 1000 seconds. As PTPMEG has no effect on LTD induction or expression in the absence of active PKC, PTPMEG was present and active throughout the 3000-second simulation.

To simulate the knockout of specific species (e.g. PTPMEG, Figs 4 and 5), these species were removed from the model.

### Sensitivity analysis

We carried out standard sensitivity analysis to measure the impact of variations in the model parameters (i.e., the reaction rates from Table 4) on the simulation results. To this end, we established the sensitivity of the steady-state plasma membrane AMPAR population *n* during LTD induction to changes in each reaction rate *r*_*i*_ from Table 4. This involved calculating the *scaled sensitivity coefficient* of *r*_*i*_ as the scaled partial derivative of the AMPAR population *n* by the reaction rate *r*_*i*_:

SSC(ri)=δnδrinri

The magnitude of the coefficient indicates the sensitivity of the AMPAR population *n* to changes in the reaction rate *r*_*i*_. The sign of the coefficient indicates whether *n* increases (*SSC*(*r*_*i*_) > 0) or decreases (*SSC*(*r*_*i*_) < 0) in response to an increase in the rate *r*_*i*_.

Table 5 shows these coefficients for the system operating with the rates shown in the supplementary material (S1 Table). Several model parameters have a small (<0.1) scaled sensitivity coefficient, indicating that the model is robust to significant changes in these parameters. The model is sensitive to the remaining parameters:

1. The rates of diffusion between the PSD and the extra-synaptic area, and between the extra-synaptic area and the endocytic zone.
2. The kinase and phosphatase kinetics, since these species are the regulators of PF-PC LTD expression as shown in the Results section.
3. The rate of exocytosis, but not the rate of endocytosis (indicating that trapping at the EZ might be rate-limiting).

**Table 5:** Sensitivity analysis results (parameters with a sensitivity coefficient of magnitude above 0.1 are emphasised in bold and discussed in the Methods section).

Parameter category	Parameter, *r*_*i*_	Scaled sensitivity coefficient, *SSC*(*r*_*i*_)
AMPAR binding interactions	k.ampar-grip.on	0.098
	k.ampar-grip.off	-0.025
	k.ampar-grip.off*	-0.049
	k.ampar-pick.on	-0.043
	k.ampar-pick.off	0.0812
	k.grip-pick.on	0.006
	k.grip-pick.off	-0.005
	k.pick-pkc.on	-0.038
	k.pick-pkc.off	-0.001
	**k.ampar-nsf.on**	**0.165**
	**k.ampar-nsf.off**	**-0.164**
Lateral diffusion	**k.diff.x-psd**	**0.703**
	**k.diff.psd-x**	**-0.675**
	**k.diff.x-ez**	**-0.650**
	**k.diff.ez-x**	**0.263**
Kinase and phosphatase kinetics	**kcat.ptpmeg**	**-0.175**
	**kcat.pkc**	**-0.362**
	**kcat.pp2a**	**0.333**
	**kcat.sfk**	**0.172**
	**km.ptpmeg**	**0.139**
	**km.pkc**	**0.352**
	**km.pp2a**	**-0.246**
	**km.sfk**	**-0.088**
Endocytosis and exocytosis	k.endo	-0.049
	**k.exo**	**0.431**

Experimental data from the literature was used to determine the values for these parameters that the model is sensitive to, as explained in the supplementary information S1 Table.

## Supporting Information

S1 Table Cerebellar LTD model initial conditions and parameters. (DOCX)

S1 Model COPASI model. (CPS)

S2 Model Alternative COPASI model. (CPS)

S1 Fig Representative results from alternative model (finite GRIP and PICK1). (DOCX)
