# Supplementary material for: A Computational Model for the AMPA Receptor Phosphorylation Master Switch Regulating Cerebellar Long-Term Depression
Source: PLoS Comput Biol. 2016 Jan 25;12(1):e1004664. doi: 10.1371/journal.pcbi.1004664 (PMC4726815; doi:10.1371/journal.pcbi.1004664)
Supplement: S1 Table — (DOCX) [file pcbi.1004664.s001.docx]

| **Species** | **Initial concentration (μM – except where stated)** | **References** |
| --- | --- | --- |
| AMPAR | 125 molecules | [1] |
| GRIP | 1.1 | [1] |
| PKC | 0.66 | [1] |
| PICK1 | 0.66 | Calibration† |
| SFK | 0.66 | Calibration† |
| PP2A | 1.1 | [1] |
| PTPMEG | 1.1 | Calibration† |

| **Parameter** | **Value** | **References/notes** |
| --- | --- | --- |
| k.ampar-grip.on | 5.5 μM^-1^ s^-1^ | [2] |
| k.ampar-grip.off | 0.3 s^-1^ | [3] |
| k.ampar-grip.off* | 70 s^-1^ | [1] |
| k.ampar-pick.on [high Ca^2+^] | 0.7 [2.8] μM^-1^ s^-1^ | [4, 5], calibration† |
| k.ampar-pick.off | 5.0 s^-1^ | [5], calibration† |
| kcat.ptpmeg | 5.5 s^-1^ | Calibration† |
| kcat.pkc | 4.7 s^-1^ | [1] |
| kcat.pp2a | 0.6 s^-1^ | [1] |
| kcat.sfk | 10 s^-1^ | [6] |
| km.ptpmeg | 7.8 μM | Calibration† |
| km.pkc | 126 μM | [7] |
| km.pp2a | 7.8 μM | [8] |
| km.sfk | 3.5 μM | [6] |
| k.grip-pick.on | 10 μM^-1^ s^-1^ | Calibration† |
| k.grip-pick.off | 5 s^-1^ | Calibration† |
| k.pick-pkc.on | 6.0 μM^-1^ s^-1^ | [5] |
| k.pick-pkc.off | 1.0 s^-1^ | [5] |
| k.ampar-nsf.on | 1 μM^-1^ s^-1^ | Calibration† |
| k.ampar-nsf.off | 5 s^-1^ | Calibration† |
| k.diff.psd-x | 0.02 s^-1^ | [3, 9] |
| k.diff.x-psd | 0.02 s^-1^ | [3, 9] |
| k.diff.x-ez | 0.02 s^-1^ | [3, 9] |
| k.diff.ez-x | 0.002 s^-1^ | [9], calibration† |
| k.endo | 0.03 s^-1^ | See Methods |
| k.exo | 0.045 s^-1^ | See Methods |
| pkc.act | 100 s^-1^ | PKC switch on |

**Table S1.** Cerebellar LTD model initial conditions and parameters

Gallimore, Aricescu, Yuzaki & Calinescu

Submembrane volume = endosomal volume = 7.5x10^-18^ L [10]

†Parameter estimated as experimentally-determined kinetics unavailable. Starting from sensible value estimates, we calibrated the unknown parameters by replicating LTD expression as observed experimentally. We then validated the model and its parameters by replicating a range of additional experimental results as detailed in Results and Methods of the main text.

1. Antunes G, De Schutter E. A Stochastic Signaling Network Mediates the Probabilistic Induction of Cerebellar Long-Term Depression. Journal of Neuroscience. 2012;32(27):9288-300. doi: 10.1523/jneurosci.5976-11.2012. PubMed PMID: WOS:000306193900017.

2. Gianni S, Engstrom A, Larsson M, Calosci N, Malatesta F, Eklund L, et al. The kinetics of PDZ domain-ligand interactions and implications for the binding mechanism. Journal of Biological Chemistry. 2005;280(41):34805-12. doi: 10.1074/jbc.M506017200. PubMed PMID: WOS:000232403900052.

3. Czondor K, Mondin M, Garcia M, Heine M, Frischknecht R, Choquet D, et al. Unified quantitative model of AMPA receptor trafficking at synapses. Proceedings of the National Academy of Sciences of the United States of America. 2012;109(9):3522-7. doi: 10.1073/pnas.1109818109. PubMed PMID: WOS:000300828200066.

4. Hanley JG, Henley JM. PICK1 is a calcium-sensor for NMDA-induced AMPA receptor trafficking. Embo Journal. 2005;24(18):3266-78. doi: 10.1038/sj.emboj.7600801. PubMed PMID: WOS:000232551600011.

5. Bolia A, Gerek ZN, Keskin O, Ozkan SB, Dev KK. The binding affinities of proteins interacting with the PDZ domain of PICK1. Proteins-Structure Function and Bioinformatics. 2012;80(5):1393-408. doi: 10.1002/prot.24034. PubMed PMID: WOS:000302541900012.

6. Kemble DJ, Wang YH, Sun GQ. Bacterial expression and characterization of catalytic loop mutants of Src protein tyrosine kinase. Biochemistry. 2006;45(49):14749-54. doi: 10.1021/bi061664+. PubMed PMID: WOS:000242516100019.

7. Gandy S, Czernik AJ, Greengard P. PHOSPHORYLATION OF ALZHEIMER-DISEASE AMYLOID PRECURSOR PEPTIDE BY PROTEIN KINASE-C AND CA-2+/CALMODULIN-DEPENDENT PROTEIN KINASE-II. Proceedings of the National Academy of Sciences of the United States of America. 1988;85(16):6218-21. doi: 10.1073/pnas.85.16.6218. PubMed PMID: WOS:A1988P781800094.

8. Bhalla US, Iyengar R. Emergent properties of networks of biological signaling pathways. Science. 1999;283(5400):381-7. doi: 10.1126/science.283.5400.381. PubMed PMID: WOS:000078067000046.

9. Borgdorff AJ, Choquet D. Regulation of AMPA receptor lateral movements. Nature. 2002;417(6889):649-53. doi: 10.1038/nature00780. PubMed PMID: WOS:000176001200048.

10. Kim M, Park AJ, Havekes R, Chay A, Guercio LA, Oliveira RF, et al. Colocalization of Protein Kinase A with Adenylyl Cyclase Enhances Protein Kinase A Activity during Induction of Long-Lasting Long-Term-Potentiation. Plos Computational Biology. 2011;7(6):18. doi: 10.1371/journal.pcbi.1002084. PubMed PMID: WOS:000292381900028.
