# Supplementary material for: A Computational Model for the AMPA Receptor Phosphorylation Master Switch Regulating Cerebellar Long-Term Depression
Source: PLoS Comput Biol. 2016 Jan 25;12(1):e1004664. doi: 10.1371/journal.pcbi.1004664 (PMC4726815; doi:10.1371/journal.pcbi.1004664)

**Supplementary Information.**

**“*A Computational Model for the AMPA Receptor Phosphorylation Master Switch Regulating Cerebellar Long-Term Depression*” by A Gallimore, A R Aricescu, M Yuzaki and R Calinescu**

**Representative Results from Alternative Model (finite GRIP and PICK1)**

Effect of PTPMEG on LTD inducibility.


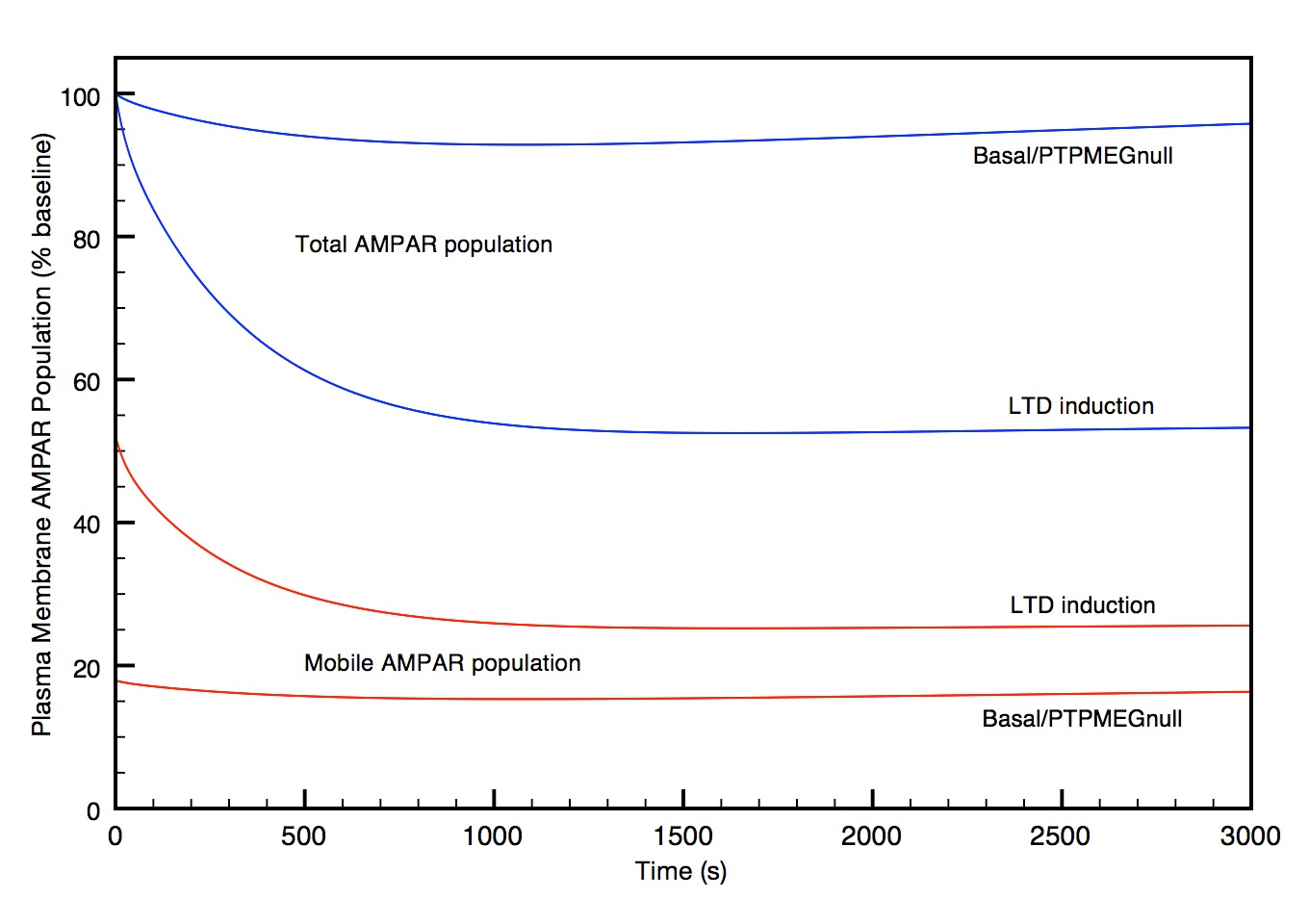


Effect of PP2A inhibition on LTD expression.


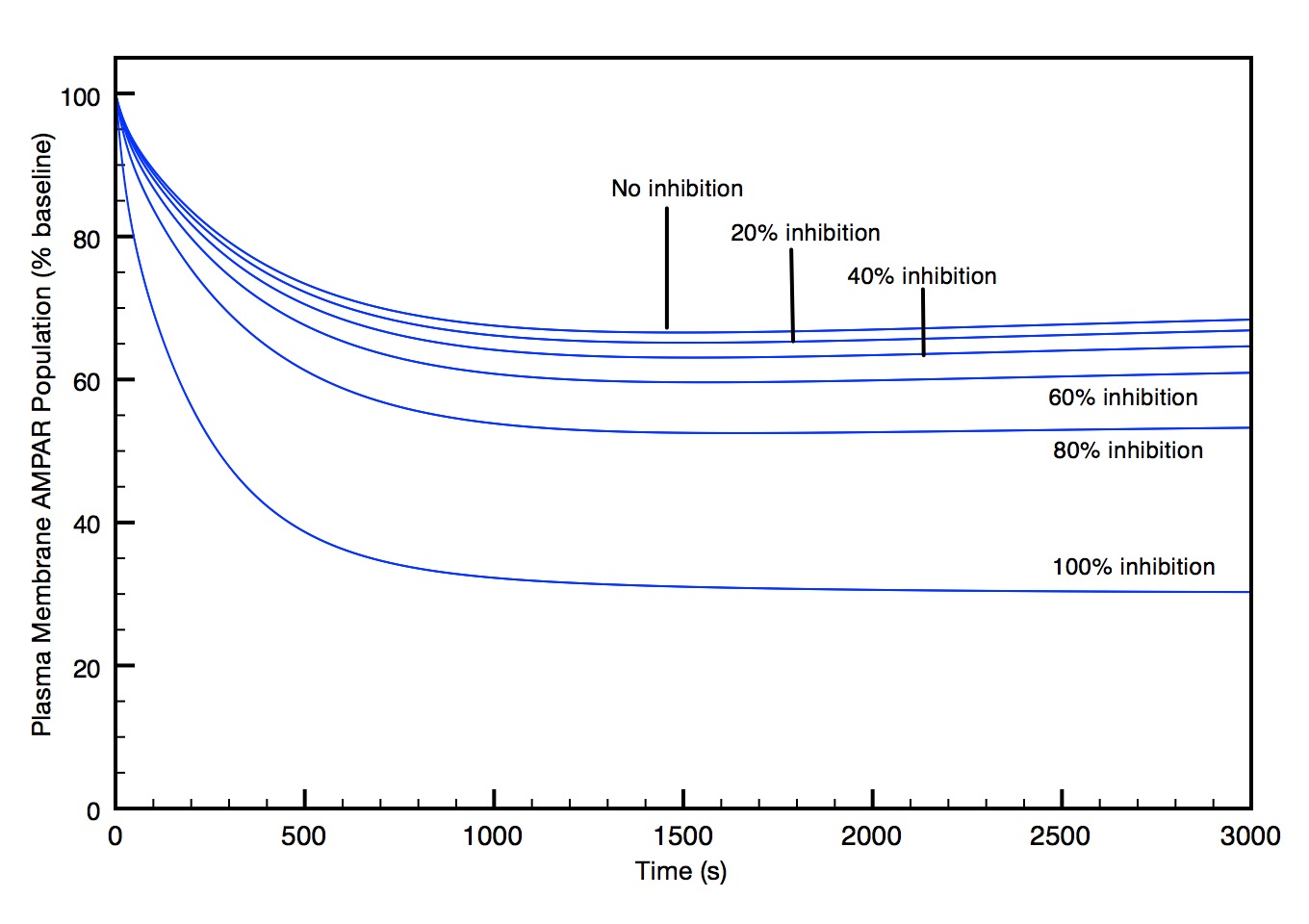


Effect of SFK concentration on LTD expression


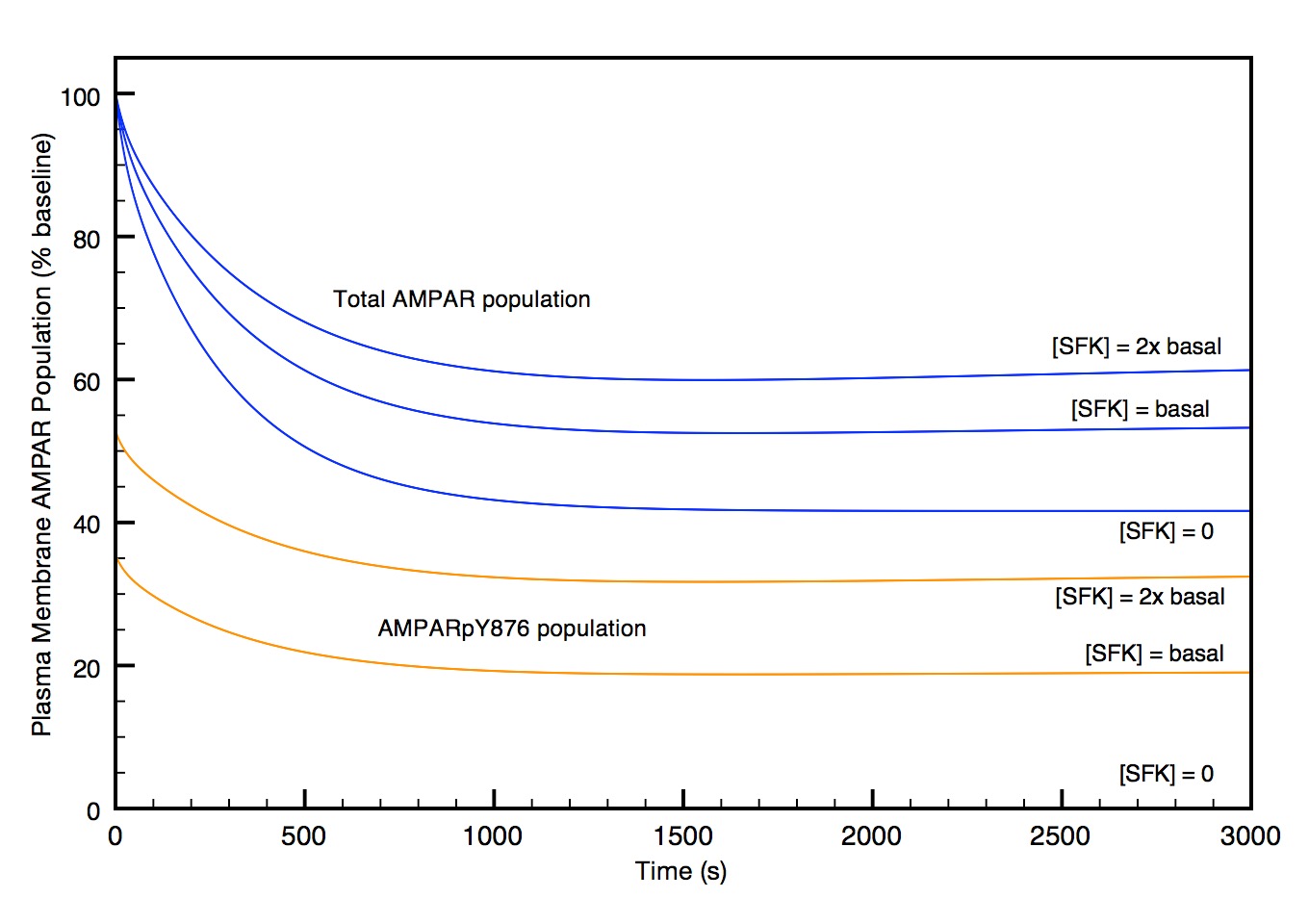

Supplement: S1 Fig — (DOCX) [file pcbi.1004664.s004.docx]
